# Gaskell, Langley, and the "para-sympathetic" idea

**DOI:** 10.7554/eLife.104826

**Published:** 2025-03-14

**Authors:** Jean-François Brunet

**Affiliations:** 1 Institut de Biologie de l’ENS (IBENS), Inserm, CNRS, École normale supérieure,PSL Research University Paris France; University of Oxford United Kingdom; Brandeis University United States

**Keywords:** autonomic nervous system, parasympathetic, sexual function, sympathetic, neuron types, anatomy

## Abstract

Historically, the creation of the parasympathetic division of the autonomic nervous system of the vertebrates is inextricably linked to the unification of the cranial and sacral autonomic outflows. There is an intriguing disproportion between the entrenchment of the notion of a ‘cranio-sacral’ pathway, which informs every textbook schematic of the autonomic nervous system since the early XX^th^ century, and the wobbliness of its two roots: an anatomical detail overinterpreted by Walter Holbrook Gaskell (the ‘gap’ between the lumbar and sacral outflows), on which John Newport Langley grafted a piece of physiology (a supposed antagonism of these two outflows on external genitals), repeatedly questioned since, to little avail. I retrace the birth of a flawed scientific concept (the cranio-sacral outflow) and the way in which it ossified instead of dissipated. Then, I suggest that the critique of the ‘cranio-sacral outflow’ invites, in turn, a radical deconstruction of the very notion of a ‘parasympathetic’ outflow, and a more realistic description of the autonomic nervous system.

## Introduction: A question of nomenclature

Langley devoted several texts to the question of nomenclature. Two of his enduring legacies in this matter are the terms ‘autonomic’ for the entire system of nerves (previously ‘visceral’ or ‘involuntary’); and ‘parasympathetic’ for one of its three divisions (alongside sympathetic and enteric). The first chapter of his 1921 monography (written in 1919) (Langley, 1921) is entitled “The divisions of the autonomic nervous system. Nomenclature”, and 7 years earlier he had already published a “Nomenclature of the sympathetic and of the related systems of nerves” in which he remarked on the difficulties of such an endeavor (Langley, 1913):

“According as the point of view is anatomical, physiological, or pharmacological the feature selected for connotation may be different. Consequently, the aim of any nomenclature should be to satisfy **as far as possible**^1; see ‘Notes’^ these several points of view.”

But all things considered, including a proposal by Heubner^2^ to create a ‘consensual’ parasympathetic system that would include innervation to the sweat glands, on account of a common mimicry by pilocarpine, Langley insisted on a nomenclature which was “almost purely anatomical” and firmly posited:

“I think the nomenclature of the nervous systems themselves should be based on anatomical facts.”

However, anatomy itself, even in its purely morphological, pre-genetic age, was never exempt from interpretation. Besides, Langley never quite stuck to his own anatomical principle and was not above reminding us (Langley, 1921, p. 8)

“that the sympathetic has, in general, opposite functional effects from those of the other autonomic nerves”

a notion contested in many details (including by Langley himself [Langley, 1900, table p. 693] — and see below) but of enduring appeal and still omnipresent in neurology textbooks (e.g. Kandel et al., 2012), despite all the qualifications; and he considered his theory “much strengthened” (Langley, 1921, p. 8) by “recent pharmacological discoveries”. And indeed, how palatable could be an anatomy which would deploy itself at odds with physiology? The stage was thus set, despite Langley’s anatomical credo, for a multiplicity of definitional criteria (anatomical, neurochemical, physiological). As it happens, they never quite fit with each other. I retrace below these fateful ambiguities concerning the ‘cranio-sacral parasympathetic’, throughout the texts of Gaskell and Langley. Lastly, I provide reasons why the field of autonomic neuroscience could benefit from foregoing the parasympathetic concept altogether.

## Gaskell’s anatomy

**Figure 1.**
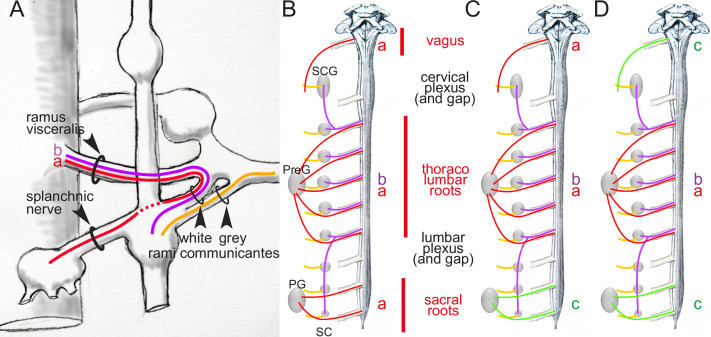
Arrangement of autonomic fibers according to Gaskell. (**A**) The three types of involuntary fibers recognized by Gaskell: the myelinated fibers (purple and red) project from the CNS, either to the paravertebral chain, through the *ramus visceralis* and white *ramus communicans* (b, purple) or, bypassing the chain (stippled line), through the splanchnic nerves to prevertebral ganglia (a, red).Unmyelinated fibers (orange) emerge from the paravertebral chain and form the gray *ramus communicans*. (**B**) Pattern of projection (adapted from Gaskell, 1920) of involuntary fibers from the CNS in three outflows, cranial, thoracolumbar, and sacral, separated by the cervical and lumbar gaps (same color code as in **A**). The rostral and caudal parts of the paravertebral chain — superior cervical ganglion (SCG) and sacral chain (SC) — receive their *rami viscerales* (b, purple) not from the adjacent level of the spinal cord, but from the thoracic and lumbar levels, respectively. Consequently, at cranial and sacral levels, all *rami viscerales* are destined to distal ganglia, and thus are of the splanchnic type (a, red). (**C, D**) Reinterpretation of the pattern by Gaskell in 1916, in the light of physiological notions: the sacral splanchnic nerves (c, green) have been dissociated from the thoracolumbar ones (**C**), and finally likened to the cranial ones (**D**) (see text for details). PG: pelvic ganglion; PreG: prevertebral ganglion.

The first and possibly most decisive act in the creation of a cranio-sacral parasympathetic system, is anatomic indeed, like Langley wanted, and actually belongs to Gaskell and his histological analysis of the course of the “connector nerves” (which we now call, after Langley, preganglionic^3^) (Gaskell, 1886). Gaskell followed these projections out of the central nervous system into the spinal nerves, as “fine medullated [myelinated]^4^ fibers” (Figure 1A). He thus called them *white rami viscerales* and noticed that they exited the spinal cord at thoracic and lumbar levels, but not immediately rostral or caudal. Rostrally, beyond a gap that corresponds (in part) to the cervical plexus, they resumed in the vagal nerve; caudally, beyond the lumbar plexus, they resumed in the pelvic nerve (Figure 1B). In the middle, at thoracolumbar level, these *white rami*

“communicate directly with the ganglia of **both** the lateral [paravertebral] and collateral [prevertebral] chains”.^5^

Those that project to the paravertebral chain form the white *rami communicantes* (Figure 1A). Those that project

“to the chain of [prevertebral] ganglia **without entering into connections** with the ganglia of the [paravertebral] chain” (p. 3)

he called ‘splanchnic nerves’ (Figure 1A). At sacral level (Figure 1B), *rami viscerales* are all ‘splanchnic’ in the sense that they

“pass directly to the prevertebral chain alone” (p. 9)

and thus

“resemble in their structure, in their method of formation, and in their passage **directly** to the [prevertebral] ganglia, **without entering** the [paravertebral] ganglia, the [myelinated] portion of the [thoracolumbar] splanchnic nerves.”

Almost 30 years later, in *The Involuntary Nervous System* (completed in 1914 but first published posthumously in 1916), the description is similar:

“[the fibers of the pelvic nerve] pass directly to [the pelvic] ganglion cells **without having anything to do** with the [paravertebral] chain**, just as** the nerves, which form the splanchnic, pass direct to the [prevertebral] ganglia of the sympathetic **independently** of the [paravertebral] ganglia” (Gaskell, 1920, p. 24)

As to the *white rami* at the cranial level, they

“can be traced **directly** into the ganglion [of the vagal trunk]”^6 ^(Gaskell, 1886, p. 11)

Thus, splanchnic nerves are similar at all levels, from cranial to sacral, in that they bypass the paravertebral chain: they “have nothing to do” with it, are “independent” of it. The thoracolumbar level stands out solely by additional projections to the paravertebral chain. Indeed, the most explicit cranio-sacral anatomical parallel in the 1886 paper is formulated as follows: the *white rami* pass

“From the sacral region into a **single stream** to the ganglia […]”
“From the thoracic region in a **double stream** […]”
“From the upper cervical region^7^ in a **single stream** […]” (p. 11)

If we label each ‘stream’ according to its nature, this [single/double/single] symmetry can be coded [a/a-b/a], *a* standing for the ‘splanchnic’ projections to distal ganglia and *b* for those to the paravertebral chain (Figure 1A and B): not too promising a start for the [parasympathetic/sympathetic/parasympathetic] arrangement of nowadays, which would be better-coded something like [c/a-b/c] (Figure 1D). What happened? Two things: two anatomic sleights of hand, drowned in the hundreds of pages of the two *J. Physiol* papers from the 1880s and *The Involuntary Nervous System* of 1916.

In 1916, Gaskell — unexpectedely, and a little reluctantly — further distinguished the sacral and thoracolumbar outflows by denying the equivalence of their respective splanchnics, despite having so explicitly likened them earlier. “Evidently”, he repeats

“the sacral outflow of fine [myelinated] fibers **corresponds in position** to those of the thoracico-lumbar *rami communicantes*, [and] for this reason, and because the name *nervus erigens*^8^ only expressed part of the function of this sacral outflow, I originally called this nerve the *pelvic splanchnic*.”(Gaskell, 1920) (p.26)

Nevertheless, Gaskell now formally proclaims his nomenclatural apostasy:

“There are many advantages in confining the use of the word splanchnic to fibers connected with the sympathetic system, so it is better to leave out the term splanchnic and call the *nervus erigens*, **as Langley has done**, the *pelvic nerve*.”

What are these “many advantages” in banishing the pelvic nerve from the company of the thoraco-lumbar splanchnics? Gaskell does not say, except, in a rather circular way, that it avoids confusing them. The circularity is broken only by the fleeting mention that the thoracolumbar is “sympathetic” and, by inference, the pelvic is not. Why not? Because we are no longer in 1886, and it is now 15 years since Langley (to whom allegiance is explicitly made) has consolidated the cranio-sacral outflow, based however not on anatomy, but on physiology (as we shall see later).

**Figure 2.**
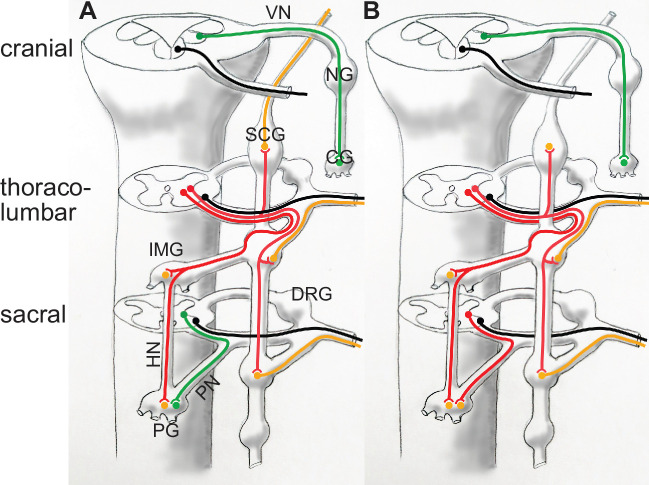
Categorization of autonomic nerves according to Gaskell and Langley (**A**), and according to genetically-defined cell types (**B**). In (**A**) the vagus nerve and the sacral splanchnic are put in the same category (parasympathetic, green) despite their projections in different types of roots: dorso-ateral (and dedicated) for the vagus, ventral (and shared with somatic motor axons, black), for the sacral, as is the case at thoracolumbar level. In (**B**) the sacral splanchnic outflow is categorized as sympathetic (red) according to its transcriptionally defined cell types, both pre- and postganglionic (Alkaslasi et al., 2021; Blum et al., 2021; Espinosa-Medina et al., 2016; Sivori et al., 2023). CG: cardiac ganglion; DRG: dorsal root ganglion; HN: hypogastric nerve; IMG: inferior mesenteric ganglion; NG: nodose ganglion; PG: pelvic ganglion; PN: pelvic nerve. SCG: superior cervical ganglion; VN: vagus nerve. Green: parasympathetic; red: preganglionic sympathetic, to both para- and prevertebral ganglia; orange: postganglionic sympathetic neurons; black: somatic motoneurons.

Now that the ‘pelvic splanchnic’ is no longer splanchnic, and that any common feature is thus erased between the thoracolumbar and sacral outflows, the original [a/a-b/a] scheme (Figure 1B) becomes more like [a/a-b/c] (Figure 1C). The last trick to arrive at the final [c/a-b/c] (Figure 1D) is to equate cranial to sacral — and so did Gaskell, following Langley’s reasoning, that we shall examine later. However, there is a serious obstacle to this, and it is anatomical: the cranial and sacral preganglionic nerves spring from different kinds of roots: ventral for the sacral (as they are for the thoracolumbar), dorsolateral for the cranial (Figure 2). But this smoking gun of the discordance between the cranial and sacral outflows, that Gaskell precisely describes (Gaskell, 1886, p. 10), was lost to him, for two reasons.

First, Gaskell mistakenly traced the white *rami* (myelinated pre-ganglionics) not only in the ventral spinal root but also the dorsal (p. 4), where they are in fact sensory (Gaskell could have seen that, unlike the ventral *rami*, they don’t disappear at lumbar and cervical levels; but concerning these fateful gaps, he somehow “confined himself” (p. 6) to a description of the ventral roots). This confusion de facto dissociated types of roots from types of nerves (the same *rami viscerales* travelling, according to him, in both ventral and dorsal roots), and comforted him in thinking that

“the exits of the different nerves from the central nervous system [are less bound up] with the true morphological meaning of the segmental arrangement of nerves [than] the arrangement of the groups of nerve cells in the central nervous system” (Gaskell, 1889, p. 153)

**Figure 3.**
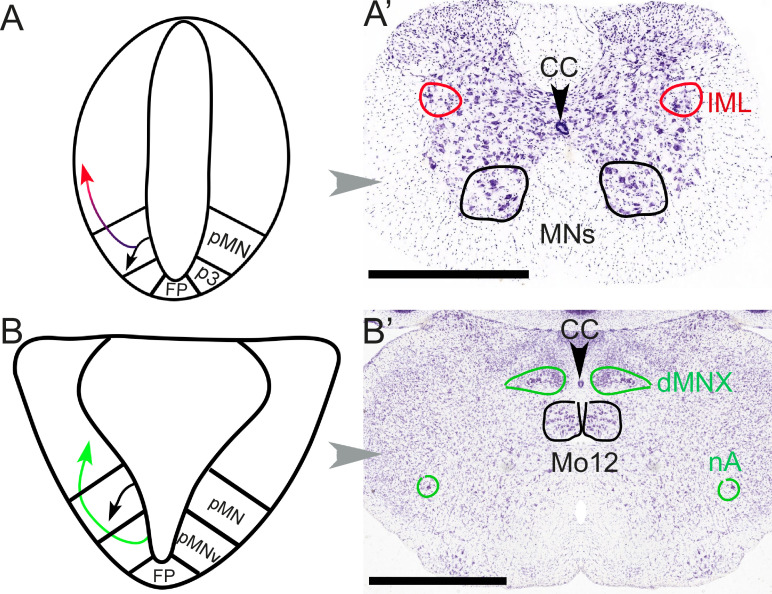
Different embryonic origins but similar migration pathways of preganglionic neurons at spinal and hindbrain levels. At spinal levels (**A, A’**) — including sacral ones— preganglionic neurons (red) arise embryonically (**A**) from the same progenitor domain (pMN) as somatic motoneurons (black), with which they share an extensive transcriptional signature, but segregate from them to settle dorsally. In the adult (**A’**), they form the intermediolateral column (improperly called ‘parasympathetic nucleus’ at sacral levels). At hindbrain levels (**B, B’**), pre-ganglionic neurons (green) arise (**B**) in a progenitor domain (pMNv) different from that of somatic motoneurons (pMN), and have consequently different transcriptional signatures. However, they also migrate dorsally (**B**) to form (**B’**) preganglionic nuclei, such as the dorsal motor nucleus of the vagus nerve. The same pMNv domain produces branchiomotor neurons (for branchiomeric muscles), as in the nucleus ambiguus (nA). CC: central canal; dMNX: dorsal nucleus of the vagus nerve; FP: floor plate; IML: intermediolateral column; Mo12: hypoglossal nucleus; nA: nucleus ambiguus; p3: progenitor domain for spinal V3 interneurons (serially homologous with the pMNv domain of the hindbrain); pMN: progenitor domain for somatic motor neurons; pMNv: progenitor domain for visceral motor neurons (bulbar preganglionics and branchiomotor neurons). Scale bars: 1 mm.

This “arrangement of the group of nerve cells” alludes to the analysis of the central nervous system in terms of longitudinal columns, that Gaskell more or less inaugurated, for better and worse. It led to the flawed notion of a continuous craniospinal visceromotor column, made plausible by the fact that both spinal and cranial preganglionic neurons are positioned (i.e. settle after a migration) dorsal to somatic motoneurons: the dorsal motor nucleus of the vagus or the nucleus ambiguus can be vaguely likened, in that respect, to the intermediolateral column of the spinal cord (Fritzsch et al., 2017) (Figure 3).

Second, and more generally, Gaskell was determined to find serial homologies between cranial and spinal nerves^9^ (see Fritzsch et al., 2017 for a more detailed account). The conceptual cost was high: it involved four components for both spinal and cervical nerves that projected in three roots in the cranial nerves, but only two in the spinal cord (Gaskell, 1889, p. 65:)^10^. Irrespective of the details, this “conception” certainly helped make “immediately vanish” “the difficulties that the homologies of the cranial with the spinal nerves had to contend with” (Gaskell, 1889, p. 65)! As C. Judson Herrick later remarked (Herrick, 1918, p. 158) “the knowledge of the composition of the cranial nerves was long retarded by persistent attempts to analyze them in accordance with the analogy of a supposed simple spinal pattern”. Ironically, Herrick’s own classification (Herrick, 1918, p. 159–162), although duly complexified by components that were “special” to the cranial nerves, was still marred by the homogenization of spinal and cranial preganglionics, directly inherited from Gaskell. At the end of the XX^th^ century, Nieuwenhuys (Nieuwenhuys et al., 1998, vol.I, p. 196) still hailed the “important contribution to the clarification of the brain stem afforded by Gaskell” and chronicled the “great impact of his ideas on a number of American neuroanatomists” (Osborn, Herrick, Strong, Johnston, etc.). Indeed, consequences of this largely misguided endeavor are still with us today, and the sacral parasympathetic is one of them^11^.

The enterprise was possibly enabled by the vertebral theory of the cranium, invented by the German Naturphilosophen of the late XVIII^th^ century and still alive during Gaskell’s youth, in his own country, under the influence of Richard Owen^12^: if the skeleton of vertebrates was a succession of fundamentally — if sometimes cryptically — similar segments, from tail to head, the hindbrain could be a seamless continuation of the spinal cord, making a cranio-sacral parallel conceivable^13^.

Yet, the distinct types of roots predictably reflect distinct types of neurons (Figure 3): the sacral preganglionics form an 'intermediolateral column', indistinguishable from the thoracolumbar one, from which it is only separated by the swelling of the lumbar motoneuronal pool, and incompletely so (Verlinden et al., 2024). Conversely, the cranial preganglionics reside in several cell groups of the hindbrain (nucleus ambiguus, dorsal motor nucleus of the vagus nerve, salivatory nuclei), quite distinct from the intermediolateral column whose rostral limit lies caudally far away, at the border of the thoracic and cervical cords. A century later we know that, unsurprisingly, the anatomic similarity between the sacral and thoracolumbar intermediolateral columns, and their difference with the cranial preganglionic nuclei, reflect their respective embryonic origins and genetic make-ups (Alkaslasi et al., 2021; Blum et al., 2021; Espinosa-Medina et al., 2016). Thus, concerning preganglionic autonomic nerves, there is nothing 'cranio-sacral'. And, logically, there is nothing cranio-sacral concerning the ganglionic relays either (Sivori et al., 2023), but this aspect was never central for Gaskell.

In summary, in terms of anatomy, the cranio-sacral parallel is predicated on two mistakes (which, for good measure, were contradictory with each other): the homogenization, against all odds, of the entire cranio-spinal visceral outflow; and the dismissal, for no reason, of the kinship between the thoracolumbar and sacral splanchnics. One can speculate that, without the resulting symmetry in the arrangement of preganglionic nerves, and the esthetic appeal of two similar outflows, cranial and sacral, framing at a distance a thoracolumbar one, there would have never been a ‘sacral parasympathetic’.

## Functional antagonism

The second and definitive act in the creation of the cranio-sacral parasympathetic is Langley’s 1899 *Address to the British Association for the Advancement of Science, Physiological section* at Dover, September 15, 1899 (published in Langley, 1899a). He calls the sacral and cranial systems “one system”, different from the “sympathetic”, based on a physiological argument. (He has yet to invent the term ‘parasympathetic’ which he will do 6 years later, in the unceremonious form of a footnote: “I use the word para-sympathetic for the cranial and sacral autonomic systems” [Langley, 1905, p. 403]).

As a physiologist, the memory of Langley has now largely eclipsed that of Gaskell, but it was not always so. After the posthumous publication of Gaskell’s *The Involuntary nervous system* (Gaskell, 1920), Langley, frustrated by the “tendency amongst English writers to consider that the main problems of the autonomic system were settled by Gaskell”, leaving to others (chiefly himself) the merit of “having filled in the gaps and completed the details of the main plan”, sent a protest to the editor of *The Lancet* (Langley, 1919)^14^. He accused Gaskell (who could no longer answer) of having surreptitiously erased all kinds of confusions from his 1886 paper and replaced them with Langley’s own clarifications, blurring the transition by a treatment that was “not historical”. Among other things, Langley claims credit for “essentially” modifying the “conception of the visceral system as a single system”:

“It is now held that the bulbar part of the “cranial outflow” and the “sacral outflow” **form one system for the gut and its appendages** (my oro-anal system) and that it is essentially different […] from the sympathetic.”^15^

**Figure 4.**
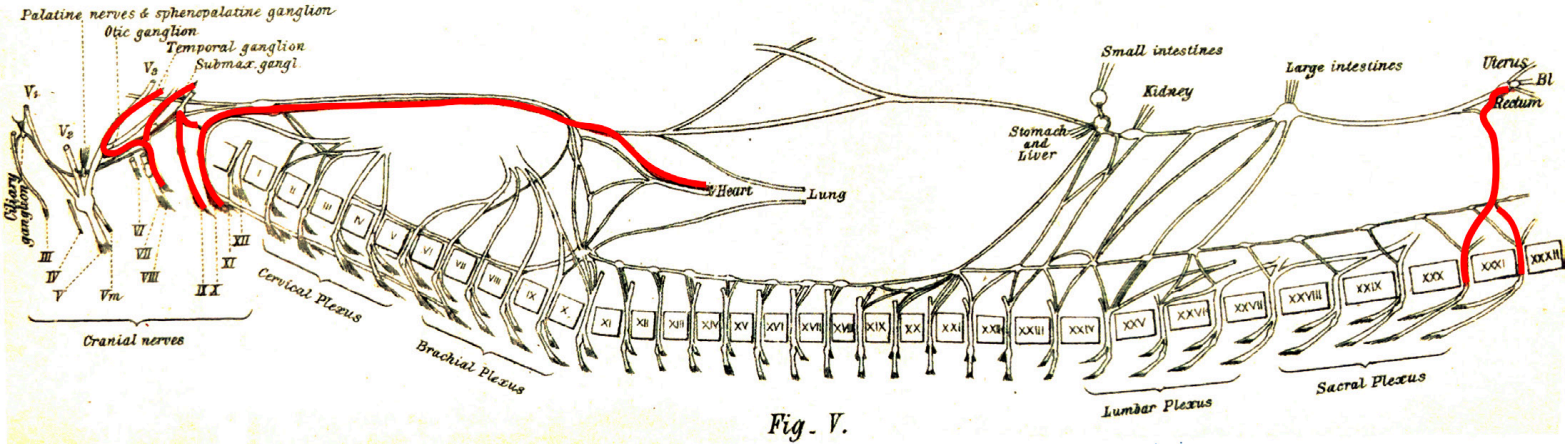
The visceral nerves of a dog as they appear in figure V, plate III, from Gaskell, 1886 (enhanced for clarity). ‘Vaso-inhibitory nerves’ are represented in red and are found at cranial and sacral levels.

But here, it seems that it is Langley who fails to give proper credit to Gaskell. If Gaskell’s 1886 anatomy was erroneous on many points (particularly the nature of various ganglia), and tentative about a cranio-sacral parallel, some of his physiological remarks prefigure Langley’s views. In particular, Gaskell explicitly likens the *nervus erigens* to the vagus, both “vasoinhibitory nerves *par excellence*” (Gaskell, 1886, p. 28), and describes the origins of the vasomotor and vasoinhibitory nerves as mutually exclusive: respectively thoracolumbar and cranio-sacral (Figure 4).

Langley was possibly justified in ignoring Gaskell’s physiological argument on account that it got muddled in *The involuntary nervous system* (in the meantime, Gaskell had found vasoinhibitory nerves also at thoracolumbar level, after all), and that Langley’s own crucial vascular argument is more specific, as I explain below.

**Figure 5.**
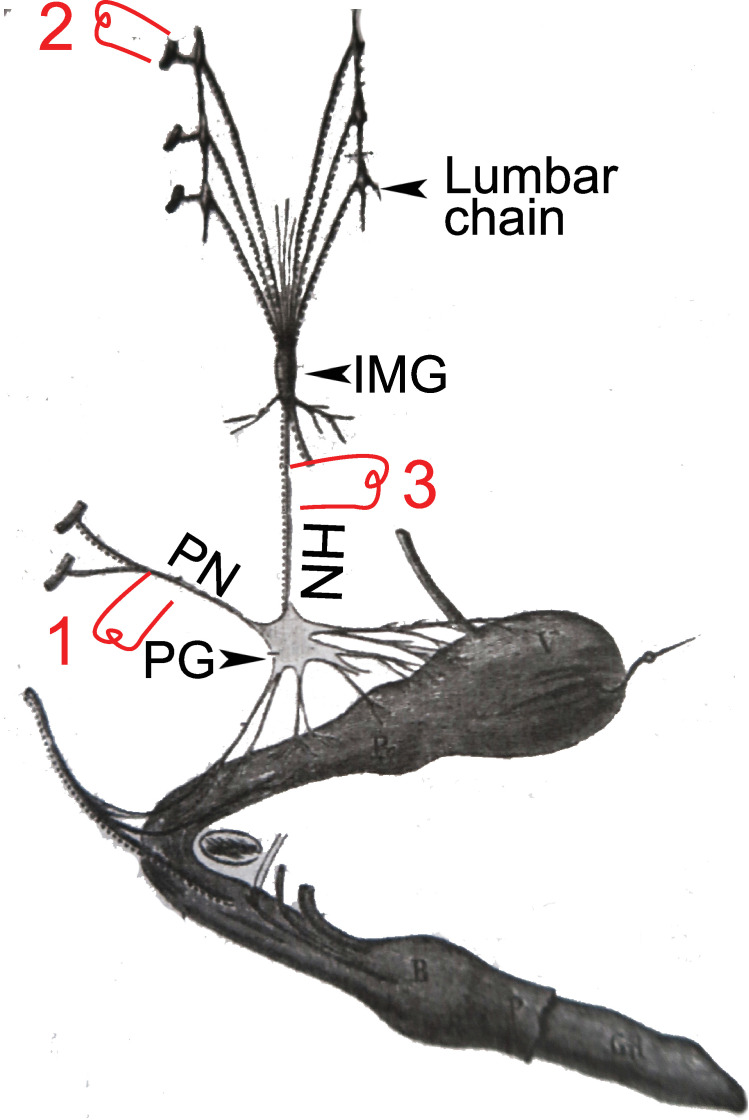
A dog’s penis and its innervation (reproduced and adapted from François-Franck, 1895). HN: hypogastric nerve; IMG inferior mesenteric ganglion (or plexus); PG: pelvic ganglion (or hypogastric plexus); PN; pelvic nerve or *nervus erigens* of Eckhardt. 1, 2, & 3: electrostimulation sites used by Langley and others, see main text for details.

In his *Address*, after mentioning the view of “some observers” that the cranio-sacral system is in fact part of a general one, only separated by the “nerve-centers for the arms and the legs”, Langley, in the founding moment of the “sacral parasympathetic”, dissents, based on “one reason” (p. 885): it concerns “certain blood vessels” that receive a double input from the lumbar and sacral levels, with opposing effects. This is an allusion (remarkably cryptic, perhaps for the sake of decency during a public discourse^16^) to his 1895 monography on the blood vessels of the “external generative organs”: the helicine arteries (terminal branches of the cavernous artery) whose dilatation after stimulation of the sacral outflow underlies erection, and constriction after stimulation of the lumbar outflow (as Langley reported) causes detumescence (Langley and Anderson, 1895) (Figure 5). And he concludes that the two outflows are different. As far as I am aware, this 1899 statement closes forever Langley’s interest in the blood vessels of the external genitals, the crux of the matter for the “sacral parasympathetic”, its “one reason”.

Let’s remind the reader of the details and fate of Langley’s physiological observation (Langley and Anderson, 1895). Langley obtained, like Eckhardt had done 30 years earlier, massive vasodilation of the helicine arteries (thus erection) upon stimulation of the pelvic nerve (1 in Figure 5), a “very great contraction” (p. 87) of the same arteries (thus detumescence) when stimulating the lumbar sympathetic trunk or its roots (2 in Figure 5), but also “some degree of contraction” (p. 103) when stimulating the hypogastric nerve — the lumbar “splanchnic nerve” which projects past the paravertebral chain and through the inferior mesenteric plexus, en route to the pelvic ganglion (3 in Figure 5). On this latter point^17^, however, Langley was contradicting previous studies, summarized in Espinosa-Medina et al., 2018 and that he duly cited and discussed (p. 85–87 and p. 103) — notably by Eckhard, 1876 and François-Franck, 1895. Thereafter, he was himself repeatedly contradicted: by Root and Bard, 1947 using lesion experiments, and, using electrostimulation like Langley himself, by Bacq, 1935; Bessou and Laporte, 1961 and Sjöstrand and Klinge, 1979, who all found a proerectile pathway in the hypogastric nerve, for which Dail proposed an anatomical substrate (Dail et al., 1986). In this time span, Semans and Langworthy, 1938 are pretty much alone in corroborating Langley’s view of an exclusively anti erectile role of the lumbar spinal cord. The current general view (sometimes reticent or buried in the fine print) is that there is indeed a lumbar (thus sympathetic) pro-erectile pathway, traveling in the hypogastric nerve, which is revealed either by its electrical stimulation, or by destruction of the sacral pathway (experimentally in animals, accidentally in humans^18^). The reason why Langley missed it has been attributed (Bessou and Laporte, 1961; Root and Bard, 1947) to the coexistence of dilator and constrictor fibers in the hypogastric nerve, the latter masking the former in some stimulation paradigms. Incidentally, such coexistence might be found also in the first sacral root of the pelvic nerve which, in the hands of François-Franck, 1895, could elicit either dilation or constriction, as noted by Langley (Langley and Anderson, 1895, p.86). All in all, the erectile function is “well duplicated” between lumbar and sacral levels (Jänig, 2006) (p. 357): the antagonism across this anatomic boundary in the spinal cord — the *only* physiological reason that Langley ever gave for placing the sacral outflow in the parasympathetic system — does not exist.

How, then, could the ‘sacral parasympathetic’ survive to this day? How the many proponents of two pro-erectile pathways, one lumbar and one sacral, could keep calling them, respectively, ‘sympathetic’ and ‘parasympathetic’, when the latter was christened explicitly to signify that this function was not shared by the former?

A reason might be that, when the first contradiction to Langley was published in the late 1940s (Root and Bard, 1947) —arching back to Eckhardt and François-Franck—, the authors did not remember the origin of the term “para-sympathetic”, hidden in a footnote from Langley, 1905 (p. 403), which does not even refer to the 1895 study that inspired it (Langley and Anderson, 1895). Another reason might be that, in this interval, the discovery of the chemical neurotransmitters noradrenaline and acetylcholine had finally explained functional antagonism (whose mechanism remained elusive for Langley^19^) thus consolidating the general notion of two antagonistic autonomic outflows and, so to speak, emancipating the term ‘parasympathetic’ from its (erroneous) sacral origins. A final irony however, is that, concerning erection, the chief antagonist of noradrenaline is not acetylcholine, but nitric oxide, whose proerectile role would not be established before another half-century (Burnett et al., 1992), which has nothing to do with the “receptive substances” discovered by Langley, and which played no role in establishing the classical view of the autonomic nervous system that we inherited from him.

To this day, the anatomical substrate of the neural antagonism on the helicine arteries, thus erection, is uncertain. For all we know, it might play out between the sacral paravertebral chain and the pelvic ganglion (Vanhatalo et al., 1996): since the former receives input from the lumbar cord exclusively (Figure 1B), and the latter from both, the lumbar and the sacral cord (Sivori et al., 2023), this would represent, at the level of the spinal cord, a lumbar/lumbo-sacral antagonism. And little is known of the projections from higher brain centers on the lumbar and sacral spinal cord which are paramount to trigger or terminate erection — too often, and not very aptly, called a ‘spinal reflex.’ The physiology also is poorly understood: it could consist in a pseudo-antagonism (as in the case of the iris, see below), whereby the variations in the diameter of arteries depend exclusively on fluctuations of the lumbo-sacral proerectile pathway, against the background of a weak noradrenergic tone, as suggested by Sjöstrand and Klinge, 1979. The idea of a ‘sacral parasympathetic’, by appearing to offer easy answers to all these questions, might be partly responsible for our current lack of understanding.

Another adverse long-term effect of the (non-existent) lumbo-sacral antagonism on the helicine arteries — and an unwitting part, this time, of Langley’s legacy — is that by echoing the (undisputed) cranio-thoracic antagonism on the heart, it only helped planting the notion that the sympathetic and parasympathetic systems function antagonistically *in general*, the didactic basis of all presentations of the autonomic nervous system to this day. A detailed criticism of this exaggeration, or oversimplification is outside the scope of this review, but I will just mention the degree to which it was peripheral to Langley’s views.

In the 1899 *Address*, strangely, he did not even bother to mention, alongside the functional lumbo-sacral antagonism that he put forward, the thoraco-cranial one on the heart, which is required to justify the cranio-sacral parallel: perhaps he was loathe of treading on Gaskell’s turf; or he counted that this fact was sufficiently on his audience’s mind. At any rate, he never worked himself on the heart (which remains to this day the canonical example of a *bona fide* antagonism). Langley never touched any antagonism on the iris, where he only studied the sympathetic input (to argue for the existence of an atypical 'dilator muscle' controversial for a long time) (Langley and Anderson, 1892)^20^. Concerning the bladder, a major conclusion of his monography (Langley and Anderson, 1895) had been that:

“We give some additional—and we think conclusive—evidence that **both the lumbar and the sacral nerves cause contraction** of all the muscle fibers of the bladder […]. Inhibitory fibers of the bladder are few, if indeed they exist.”^21^

Concerning the salivary glands, he had described the effects of sympathetic and parasympathetic innervation as both secretory, distinct only as to the quality of the saliva; any inhibition of secretion by the sympathetic — now often used as another example of sympatho/parasympathetic antagonism — he dismissed as an indirect side effect of overactivation (of dubious adaptative value, as it were):

“The sympathetic and chorda **are not antagonistic** for minimal stimuli, and the **apparent antagonistic** action of the sympathetic on the chorda for maximal stimuli is probably due to a diminution in blood supply.” (Langley, 1878)

Most strikingly, despite the lone general statement, already cited, that

“the sympathetic has, **in general**, opposite functional effects from those of the other autonomic nerves” (Langley, 1921, p. 8)

no organ displaying an antagonistic effect of the sympathetic and parasympathetic systems features in the whole of *The Autonomic Nervous System (Part I*): not the heart (except paradoxically, to suggest that there is no dual innervation of the ventricle in some species, p.20), not the bladder, not the iris, and certainly not the blood vessels of the external genitals. The fateful paragraph arguing for a physiological antagonism — lumbo-sacral, on the helicine arteries— which inaugurated the current dogma of a sacral parasympathetic outflow, is a singularity in Langley’s body of work (and dates from his early period).

## Conclusion: The polytomous autonomic outflow

Once the parasympathetic nervous system is bereft of its sacral part, which was key to its definition, what remains of it? An interesting case to consider is the enteric nervous system, the largest part of the autonomic nervous system, whose inclusion or not in the parasympathetic division is the subject of a century-long hesitation. In 1899, before he had coined the term ‘parasympathetic’, Langley talked of

“The cranial and sacral systems [that] supply the muscular coats of the alimentary canal and certain structures connected developmentally with anterior and posterior portions of it. They are especially connected with these terminal portions; they send numerous nerve fibers to them, whereas they send but few to the intervening portion.” (Langley, 1899a)

a statement that included all central projections to the gut in the “oro-anal system”, despite their relative scarcity in the middle. Gaskell followed through with his “bulbo-sacral or enteral system” (Gaskell, 1920, Figure 5, p. 25). However, 20 years later, Langley chose to exclude the enteric nervous system, for one reason: the existence of the preganglionic fibers was not settled (Langley, 1921, p. 9); consequently, he put it in a class by itself. But the preganglionic fibers do exist, after all (those “few to the intervening portion”, i.e., projections of the dorsal motor nucleus of the vagus nerve to enteric neurons), and they are indistinguishable so far from other parasympathetic fibers. For this reason and others, and despite Langley’s caution, the enteric nervous system is invariably included in the parasympathetic division in schematics of the autonomic circuits. More recently, as enteric neurons revealed their diversity, experts have arched back to Langley’s late view that “the enteric nervous system is neither sympathetic nor parasympathetic but a third autonomic division” (Rao and Gershon, 2018); the Langleyan rationale being that “most of its component neurons lack direct innervation”: Langley saw the glass as possibly empty, and it is only partly full. The enteric nervous system is thus described as “*receiving* sympathetic and parasympathetic input” (Rao and Gershon, 2018). But this symmetrical formulation glosses over a fundamental anatomical asymmetry: no spinal thoraco-lumbar (sympathetic) neuron is preganglionic to enteric ones (which might receive a *ganglionic* sympathetic input, e.g. Stebbing et al., 2001; Furness, 2012), while a parasympathetic nucleus (the dorsal motor nucleus of the vagus nerve) is preganglionic to a fraction of them. Consequently, in the non-parasympathetic view of the enteric nervous system, those vagus efferent fibers which synapse onto enteric neurons (like others do onto lung or pancreatic ones), should also be removed from the parasympathetic nervous system; or else, enteric neurons that receive these inputs should be removed from the enteric nervous system, either way leading to a rather convoluted anatomy. One could add that the esophageal and gastric parts of the enteric nervous system form exactly like parasympathetic ganglia (from nerve-associated neural crest, a.k.a. Schwann Cell Precursors, in this case of the vagus nerve) (Espinosa-Medina et al., 2017), and that, like them, they largely depend on their preganglionic innervation for function (Furness, 2006). Finally, the view that the enteric nervous system does not belong to the parasympathetic robs the latter of half of its ‘rest and digest’ functions— which might explain why the “separate but equal classification of the enteric nervous system still surprises doctors and even neuroscientists” (Gershon, 1999, p. 16). I suggest that the debate cannot be settled, and is pointless, because one of its terms, the parasympathetic, is ill-defined in the first place.

**Figure 6.**
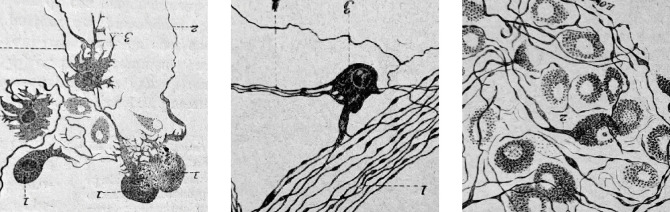
Cardiac ganglionic neurons of three morphological types recognized by Dogiel (reproduced in Testut, 1911, after Dogiel 1894). Testut provides tens of references and comments: “Despite this body of work, the question of cardiac ganglia is far from settled, owing in part to the difficulty of the subject”. Not much has happened since, in the description of cardiac neuron types.

Two other traditional components of the parasympathetic are the midbrain pathway to the eye, and the medullary (‘bulbar’) one to the heart. In both cases, knowledge of cell identities and circuitry is scant. In the case of cardiac ganglia, a great heterogeneity of cell types was noted as early as the XIXth century, by Remak, Dogiel, and many others (Figure 6), and have inspired, in more recent accounts, to call cardiac ganglia “a little brain in the heart” (Armour, 2008) — in an echo of the ‘second brain’ in the gut (Gershon, 1999) — which, like the latter, receives inputs on only a fraction of its cells McAllen et al., 2011 and displays activity after decentralization (Ardell et al., 1991).

As to the midbrain pathway to the eye, Langley himself was uneasy about its classification and declared it

“**clearly distinct** from the rest of the cranial outflow” (Langley, 1921, p. 8)^22^

**Figure 7.**
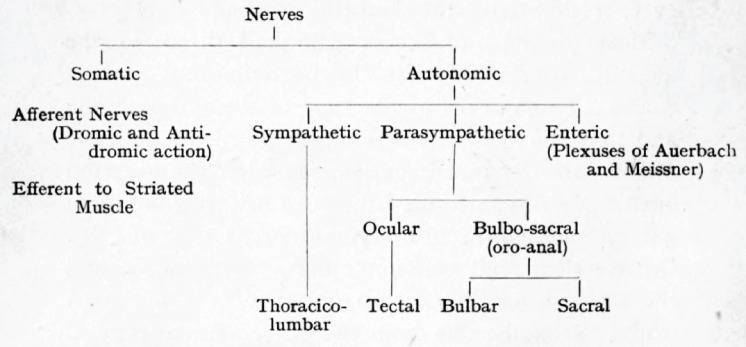
Langley’s cladogram of the peripheral nerves as it appears in *The Autonomic nervous system Part I*. The “bulbo-sacral” outflow is unified across an anatomical gap that spans most of the spinal cord, but the ocular (or tectal) is in a separate subclass, despite being cranial, like the bulbar.

He never explained in what way, and why more so than the sacral outflow (Figure 7) despite its greater anatomical proximity. But “clearly distinct” it is, indeed: for one thing, its actions are unrelated to homeostasis and only adapt a sense organ to what it senses of the outside world (light) (while the sympathetic outflow to the eye has no documented role in that); some of these actions are even partly voluntary (during accommodation to distance); and in line with its idiosyncratic roles, its development is quite distinct from that of the bulbar pathway (Sivori M and JFB, unpublished data).

Thus, it emerges that if Langley was clairvoyant in distinguishing, in the autonomic outflow, a thoracolumbar part and reserving the old term “sympathetic” for it, he was less inspired in unifying what is *not* thoracolumbar, thus sympathetic, under the umbrella of the neologism “para-sympathetic” (or even para-sympathetic+enteric). The parasympathetic nervous system — unlike the sympathetic — is not a natural kind. Rather, it is a patchwork of autonomic pathways unrelated in terms of anatomy, cell types, and physiology (one of which, the sacral outflow, or rather, the lumbo-sacral outflow to the pelvis, is actually sympathetic-like; Espinosa-Medina et al., 2016; Sivori et al., 2023). It might be fruitful to forego the Langley of 1899, who inaugurated the dichotomous age of the autonomic nervous system (notwithstanding the later provision about the enteric), and revive the Langley of 1898 who, still a pluralist, talked of

“The sympathetic and **other related systems** of nerves” (Langley, 1900)^23^

**Figure 8.**
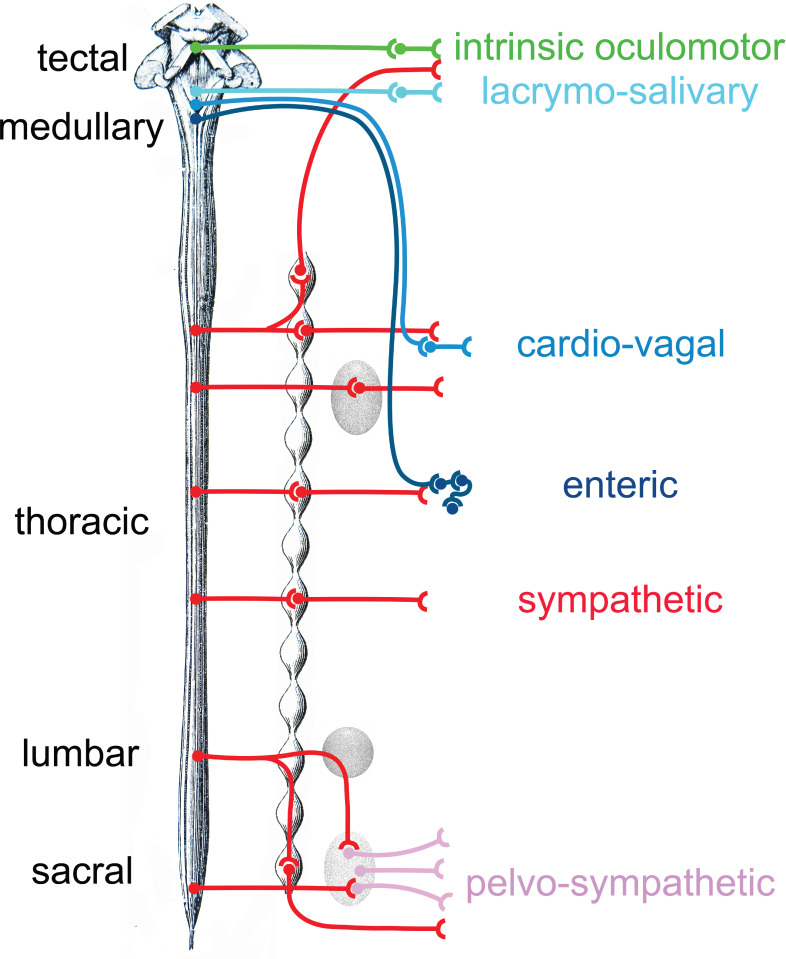
Provisional schematic of the polytomous, or mosaic autonomic outflow, based on published data (for the lumbosacral outflow, which was tentatively named ‘pelvosympathetic’; Sivori et al., 2023), unpublished data (for the oculomotor outflow), and educated guesses (for cardiac ganglia). The lumbo-sacral outflow is distinguishable from the sympathetic (red) mostly at the level of post-ganglionic neurons, in the pelvic ganglion (pink). The genetic differences between the three medullary outflows (lacrymo-salivary, cardio-vagal, and enteric) are hypothetical at the preganglionic level (i.e. between the salivatory nuclei, the dorsal nucleus of the vagus nerve and the nucleus ambiguus). Note that the six outflows that are distinguished in this provisional scheme (including the pelvosympathetic one) form a series along the rostro-caudal axis of the central nervous system, restoring a link between development, cell types, and physiology.

This polytomous (or multipartite, or mosaic) view (Figure 8) of the former ‘parasympathetic’, and thus of the entire autonomic nervous system, has the potential to enable two research agendas.

First, from an anatomical and physiological viewpoint, it provides realistic intermediate classification levels of neuron types, within which finer-grain parcellation can be made (e.g. on the basis of connectivity). Neuron types are organized hierarchically, at least on a regional scale (Zeng and Sanes, 2017), each level of the hierarchy corresponding to a set of common features. There are several types of cortical pyramidal cells, or of spinal motoneurons, but they remain all pyramidal cells or spinal motoneurons, with shared differential traits. In the same way, there are several types of sympathetic ganglionic neurons (Furlan et al., 2016; Wang et al., 2025) but comparison with other autonomic ganglia reveals that they are all meaningfully grouped as sympathetic ganglionic neurons — and actually difficult to parse on a genetic basis (see the Uniform Manifold Approximation and Projection in Sivori et al., 2023). Concerning the autonomic preganglionic neurons, their transcriptomes are yet to be directly compared, but what we know of their respective embryonic origins (Figure 3) and genetic determinants makes it likely that the 15 subtypes of spinal preganglionics (Alkaslasi et al., 2021; Blum et al., 2021) will group together, away from cranial ones. By contrast, there is no parasympathetic neuron class, pre- or post-ganglionic, that correspond to the “parasympathetic nervous system” as currently defined (Espinosa-Medina et al., 2016; Sivori et al., 2023 and references within). Rather, it is each branch of the “related systems of nerves” that is likely to correspond to meaningful classes of neurons.

Second, the polytomous view should set the phylogenetic agenda — so dear to Langley, and legitimately so, regardless of his fanciful scenarios^24 ^— on a firmer footing than the sympathetic/parasympathetic dichotomy. No evolutionary event is likely to have spawned the ‘parasympathetic nervous system’, but there is hope that each branch of the polytomous autonomic nervous system (Figure 8), with its particular developmental mode and physiological function, can be traced to a node in the Tree of Life.

## Notes

^1^ Emphases in the quotes (in bold) are mine.

^2^ Possibly the pediatrician Otto Heubner (1843-1926).

^3^ i.e. that project to ganglia of the “involuntary nervous system” (which in the 1880s, at the time when Gaskell was writing his foundational papers, were of uncertain status, sometimes seen as “nutritive centers” for the nerves).

^4^ I occasionally add a contemporary term, or substitute it, in brackets.

^5^ The classification of ganglia by Gaskell, 1886, conceived before the neuron theory of Cajal, let alone the distinction by Langley between preganglionic and postganglionic fibers, and later the discovery of cranial sensory neurons, is hopelessly confusing from a contemporary perspective. Only those points which were used to create the cranio-sacral outflow deserve a mention. For this reason and for the ease of reading, I occasionally replace Gaskell’s terms by contemporary ones, in brackets.

^6^ Here Gaskell seems to imply that the jugular and nodose ganglia are in series with the prevertebral ganglia. But we now know that they are sensory.

^7^ The vagus is bulbar and not “upper cervical”, but Gaskell mistakenly includes in his analysis the branchiomotor fibers of the “spinal accessory nucleus”.

^8^ ‘erector nerve’ in latin, name given by Eckhard, 1876.

^9^ This quest, which occupies section IV of Gaskell, 1886 and the whole of Gaskell, 1889, can be viewed as the first act of the grand comparative entreprise of *The Origin of the vertebrates* (Gaskell, 1908), which juggles analogies between organs and creatures in a way that makes Geoffroy Saint-Hilaire appear sober in retrospect.

^10^ “Each spinal segment gives origin to **three roots**, viz. anterior, posterior, and lateral, combined with the further conception that these **three** roots may be grouped together **into a dual** arrangement”.

^11^ “The general visceral elements in the brain stem are considered equivalent to the cells in the lateral horn of the spinal cord” (Nieuwenhuys et al., 1998), vol. I, p. 197.

^12^ Owen published an *Anatomy of the vertebrates* in 1866 and died in 1892.

^13^ The influence that I suspect, however, must have been unconscious, and stealthily passed from the bones to the nerves, and anyway forgotten 20 years later, since (Gaskell, 1908) barely mentions Goethe, Oken or Owen himself and deems “hopeless to resolve the cranium into vertebral segments” (p. 124).

^14^ This protest suggests that *The Autonomic nervous system (Pt. I)*, written in the same year, was put together in a huff, as an effort to supersede Gaskell’s book. This would explain the clumsiness of this unlikely grand finale, much less structured than Langley’s own 1900 treatise. Indeed, after the first two chapters of nomenclature and generalities (where Langley replaces Gaskell’s “involuntary” with “autonomic”, “enteral” with “parasympathetic”, “connector” with “preganglionic”, etc.), and which is probably all that people read nowadays, it is difficult to pick one’s way among a hodge-podge of detailed historical accounts, ongoing debates, experimental minutiae and phylogenetic speculations. The only “Tissues Innervated” (chapter 5) are the melanocytes of lower vertebrates and tissues that are… not innervated! (capillaries and skeletal muscle). Langley probably felt that he had made his point and, in the six years that were left of his life, did not bother to provide a “Part II”, whose plan would not have been easy to devise.

^15^ About Langley’s mention of “one system for the gut”, as well as the de facto exclusion of the tectal outflow to the eye from this formulation of the parasympathetic, see Conclusion.

^16^ In the slightly altered version of his address published by *The Lancet* (Langley, 1899b), Langley humorously comments, after a quote from an XVIIIth century doctor about his Majesty’s spleen, gall bladder and duodenum: “In these days we are, perhaps, less free in our allusions to our component parts”.

^17^ And on this latter point only: to the best of my knowledge no one ever questioned that the stimulation of the lumbar roots, which run in great part to the paravertebral chain, or the paravertebral chain itself, triggered vasoconstriction — an experiment also performed by Semans and Langworthy, 1938.

^18^ The pure ‘lumbar’ erection of men whose sacral spinal cord is destroyed is singled out as “psychogenic” (e.g. Krassioukov and Elliott, 2017) (but every erection is strictly dependent on a psychological trigger — or at least supraspinal, as in sleep-related erections); and its poor quality is highlighted (but it could entirely be due to the lack of sensory feedback, which is sacral).

^19^ As strange at it may sound to the contemporary reader, even though Langley discovered “receptive substances” — our neurotransmitter receptors — he never envisioned endogenous ligands for them, thus chemical neurotransmission (Vallenstein, 2006). He conceived autonomic nerves as largely equivalent, “indifferent” in their action (see the *Address*, p. 891). He was inspired in this view by his observation that a heterotopic graft of the proximal end of a cut vagus nerve onto a sympathetic ganglion was functional, enabling the vagal nerve to have sympathetic effects on the iris (Langley, 1897). For him, the effect of autonomic nerves depended solely on the nature of their targets (which was revealed by drug action). This view prevented any mechanistic explanation for a functional antagonism of different nerves on the same target, a topic with which he struggled on and off over 20 years (discussed for example in Langley, 1905, p. 403, or in Langley, 1921, p. 45). This conceptual deadlock may have played a role in Langley’s tendency to shun the idea of antagonism (see below).

^20^ Let’s remark here that the classical notion of sympatho-parasympathetic antagonism on the iris, however useful in ophtalmological practice, obscures rather than illuminates the mechanism of the pupillary light reflex, which depends exclusively on modulations of the midbrain input (Clarke et al., 2003; Lowenstein, 1950). Morevoer, given that the ciliary muscle receives no sympathetic innervation, such antagonism cannot have any relevance for accommodation to distance.

^21^ Another twist in this story, which would deserve another study, is that many after Langley, inspired by his idea of a lumbar sympathetic/sacral parasympathetic dichotomy, looked for a lumbo-sacral antagonism on the bladder — and occasionally even found it, as is often the case of desirable outcomes — thus contradicting Langley himself. But, despite most summary diagrams, such an antagonism is negligeable and probably nonexistent in many species, including humans (e.g. Fowler et al., 2008).

^22^ And see note 15.

^23^ This text, published in 1900 but written in 1898, is credited in Jänig, 2006 (p. 168) for presenting the first classification of the enteric nervous system as a separate division, but in fact Langley had not yet invented the parasympathetic, and he treated the sacral and tectal outflows also separately, among the “related systems of nerves”.

^24^ Langley frequently described the autonomic divisions in terms of development and evolution (this combination, which could appear as a visionnary ‘evo-devo’ synthesis, was common place at a time when the Haeckelian recapitulation still held sway over embryological thinking). This evolutionnary line of thought is already present in the 1899 Address (Langley, 1899a), in the form of what sounds nowadays almost like a non-sequitur: right after the physiological statement that the lumbar and sacral outflows have opposite effects on the helicine arteries, rather than simply concluding that they are different, Langley writes:

“Thus, it seems to me probable that in the **evolution** of mammals, the sympathetic nerves have **developed at one time**, and the cranial and sacral involuntary nerves have **developed at another time**.”

And several pages of Langley, 1921 are devoted to a ‘parasympathetic first’ evolutionnary scenario, which I do not have the space to recount here.
